# Acupuncture and Moxibustion for restless legs syndrome: A systematic review and meta-analysis protocol

**DOI:** 10.1097/MD.0000000000018827

**Published:** 2020-01-24

**Authors:** Zhijun Huang, Cao Qingqing, Zhang Wenchun, Wu Zhouhang, Ren Jiankun

**Affiliations:** a Jiangxi University of Traditional Chinese Medicine, Nanchang, Jiangxi; b Henan Vocational College of Nursing, Henan, China.

**Keywords:** acupuncture and moxibustion, meta-analysis, restless legs syndrome, systematic review

## Abstract

**Introduction:**

Previous reviews indicate that the effect of acupuncture and moxibustion (AM) on restless legs syndrome (RLS) remains uncertainty. The results of trials published in the past 12 years may possibly change this situation, but an updated systematic review is not available. We therefore designed this study to systematically assess the effectiveness and safety of AM for treating RLS.

**Methods and analysis:**

Nine online databases will be searched from inception to October 01 2019; there will be no language restrictions on the included trials. Randomized controlled trials that included patients with RLS receiving AM therapy versus a control group will be included. The selection of studies, risk of bias assessment and data extraction will be conducted by 2 independent researchers. Data synthesis will be performed by using RevMan V.5.2 software with fixed effects model or random effects model, according to the heterogeneity test. The dichotomous data will be presented as risk ratios with 95% confidence intervals (Cis) and the continuous data will be presented as weighted mean differences or standardized mean differences with 95% CIs. Evidence quality will be evaluated by using the grading of recommendations assessment (GRADE), development and evaluation system with low risk, unclear risk, and high risk.

**Ethics and dissemination:**

This systematic review and meta-analysis is literature research which will not refer to private information and not impair one's health, so, ethical approval is not required. The results of this study will be published in a journal or concerned conferences.

**PROSPERO registration number:**

CRD42019148325

**Strengths and Limitations of this study**

1) Although systematic reviews of acupuncture for RLS have been conducted previously, this study will update the evidence base by including many clinical trials that have been published in the past 12 years.
2) Evidence quality will be evaluated using the GRADE system, which would help clinicians and patients with RLS decide whether to choose AM therapy.
3) As several clinical outcomes have been used in published trials, a pooled analysis of all included studies may not be possible; however, subgroup analyses will be performed according to different outcomes.

## 1 Introduction

Restless leg syndrome (RLS) is defined as an irresistible 2-legged jitter, often accompanied by at least 2 perineodynias or hyposensitivity, which can be partially or completely relieved by activity.^[1–7]^ Patients often use pain, tingling, crawling, burning, electric current or nervousness to describe their feeling of discomfort.^[8]^ The frequency, duration, and severity of RLS symptoms vary,^[9]^ often accompanied by periodic leg movements and autonomic symptoms.^[9]^ RLS has a major negative impact on a patient's mood, health, work, and social activities,^[10]^ and sleep disruption and daytime fatigue caused by RLS are often the most common causes of patients seeking medical attention. The prevalence of RLS varies significantly across countries and geographies. Epidemiological studies have shown that the prevalence of RLS in adults (age 18 or older) ranges from less than 1%^[11]^ in Singapore to about 10%^[12]^ in Europe and the United States. This difference may be due to differences in research methods. Ethnic and genetic factors may also play a role in this difference.^[13]^

There are no specific laboratory indicators to confirm the diagnosis of RLS. The diagnosis depends mainly on the detailed clinical history. Commonly used auxiliary examinations include polysomnography and suggestive braking tests. From the first diagnosis in 1960,^[14]^ to the latest diagnostic criteria developed by the International Restless Leg Syndrome Study Group in 2014,^[9]^ the diagnosis requires patients to meet the following 5 criteria:

(1) Activities The strong desire of the legs, often accompanied by discomfort in the legs, or the feeling of discomfort in the legs leads to the desire for activity;
(2) The symptoms appear or aggravate during rest or inactivity, such as lying or sitting;
(3) The symptoms are obtained during the activity or complete relief, such as walking or stretching the legs;
(4) Symptoms in the evening or night, or only in the evening or night;
(5) The above clinical symptoms can not be explained by another disease or behavioral phenomena (such as muscle pain, legs Departmental discomfort, discomfort, varicose veins, lower extremity edema, arthritis, habitual leg shaking).

The cause of RLS is not fully understood. Iron deficiency, renal failure, and pregnancy may be 1 of the causes of RLS and are therefore considered secondary RLS.^[15]^ In addition to the above identified causes, there are no known physical abnormalities associated with the disease.^[16]^ The primary RLS etiology hypothesis is related to brain iron homeostasis.^[12,17,18]^ A study using magnetic resonance imaging showed a decrease in substantia nigra and chitin iron levels in patients with primary RLS.^[19]^ Interestingly, iron is a cofactor for tyrosine hydroxylase, the rate-limiting enzyme in dopamine production.^[20]^ Although the exact role of dopamine in the pathogenesis of RLS remains unclear,^[21]^ some double-blind clinical trials have demonstrated the use of the dopamine precursor levodopa (L-dopa) or dopamine agonist,^[17,22,23]^ suggesting that both dopaminergic system dysfunction and brain iron homeostasis may lead to RLS.^[17]^

The Restless Leg Syndrome Foundation Medical Advisory Board^[24]^ recommends l-dopamine agonists as first-line drugs for the treatment of RLS. Dopamine agonists are less likely to cause enhancement and rebound. Enhancement was defined as early onset of RLS symptoms during the day, rapid onset at rest, dopaminergic treatment aggravated symptoms, and shorter symptom relief.^[20]^ Rebound is a symptom of RLS that occurs when the drug's effect is gradually weakened.^[25]^ In 2005, ropinirole, a non-ergoline-based dopamine agonist, became the only drug approved by the US Food and Drug Administration for the treatment of moderate to severe RLS.^[17]^

As a traditional Chinese medicine therapy, acupuncture and moxibustion (AM) has the unique capability of performing holistic treatment, and some studies suggested that it is an effective and safe therapy for RLS.^[26]^ Traditional science believes that acupuncture acts on the nervous system, neurohormones, and psychological mechanisms^[27]^ and is considered to have an analgesic effect.^[28]^ Therefore, acupuncture has the potential to be an effective treatment option for RLS. However, studies have shown that long-term, high-dose dopaminergic or dopamine agonists may cause exacerbations and/or “deterioration” during the treatment of RLS. The main manifestations are: early onset of symptoms during the day; shortened latency during rest; the symptoms continue to worsen with appropriate dopaminergic drugs or increased doses; symptoms extend to previously unaffected sites; duration of efficacy is shortened.^[29,30]^ The a-2-S calcium channel ligand causes a lower risk of exacerbation and/or worsening of the patient, but common adverse reactions include dizziness, headache, lethargy, fatigue, and unstable walking.^[24,31,32]^

However, from the perspective of evidence-based medicine, the efficacy of acupuncture in the treatment of RLS remains controversial. In order to assess the clinical benefits of acupuncture treatment of RLS, a systematic review has been conducted,^[33]^ but due to poor quality trials, including the most recent studies reviewed prior to 2007, are inconclusive. Recently, it has been interesting to note that at least 5 clinical studies have been published or have been roughly estimated over the past 12 years.^[34–38]^ These studies have great potential to change the evidence base for current acupuncture treatment of RLS. However, there is currently no recent systematic review or research publication on this issue. Therefore, we have a unique opportunity to re-evaluate this issue and envision this systematic review to determine the effectiveness and safety of acupuncture based on the most comprehensive and up-to-date resources of RLS patients.

## 2 Methods

### 2.1 Criteria for inclusion

The criteria are prespecified according to the PICOS criteria that refer to patient or population, intervention, comparison, outcome, and study design.

### 2.2 Types of participants

We will includ patients with primary RLS consistent with the diagnostic criteria defined by IRLSSG^[39,40]^ irrespective of gender, race, age, and setting. We excluded patients with any signs of psychiatric or organic disorders.

### 2.3 Types of interventions

The AM of the experimental group must include forms of needle insertion including fine needle, floating needle, electroacupuncture, and so on or moxibustion at acupoints or trigger points, and the experimental group also is AM plus other interventions.

### 2.4 Types of comparator(s)/control

The control group, accepted with sham acupuncture, placebo control or other active therapies, will be included. We will not limit the treatment sessions, acupoint numbers, retaining time, and frequency. However, the comparisons of different forms of AM, such as acupuncture versus moxibustion, will be excluded.

### 2.5 Types of outcome indicators

#### 2.5.1 Primary outcomes

(1) Unpleasant sensations of RLS measured by any type of validated scale (for example, visual analog scale).
(2) Improvement of overall symptoms measured as a dichotomous outcome (remission versus no remission).

#### 2.5.2 Secondary outcomes

We also considered the following outcome measures:

(1) Periodic leg movements during sleep index;
(2) Absolute or percentage reduction in RLS frequency and duration;
(3) Sleep disturbance measured on a scale (eg, sleep onset latency);
(4) Wakefulness after sleep onset or by reported total sleep time;
(5) Daytime functioning;
(6) Quality of life measures (eg, SF-36);

#### 2.5.3 Types of studies

Randomized controlled trials (RCT) will be included. Multiple arms trials met the above criteria will be included. For crossover trials, data will be extracted from the first period only, to avoid potential carryover effects. Another study design will be excluded.

### 2.6 Search methods for identification of studies

#### 2.6.1 Electronic searches

From the inception date January 01, 2020, the following databases will be searched: PubMed, Web of Science, the Cochrane Library, EMBASE, Chinese National Knowledge Infrastructure, Chinese Biomedical Literature Database, Wanfang Database, the Chongqing VIP. The searching strategy of PubMed is presented in Table 1.

**Table 1:**
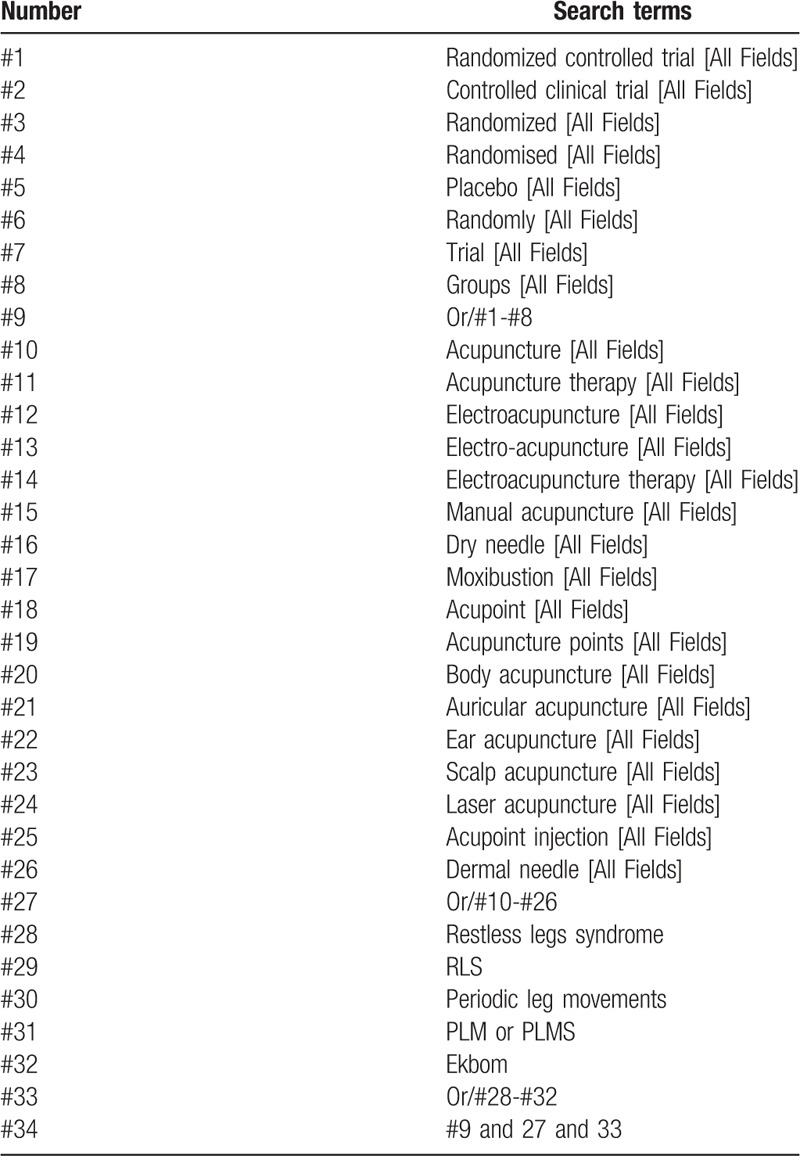
Illustrate the terms and logic of search strategy; Table 1 Search strategy used in Pubmed database.

#### 2.6.2 Searching other resources

Unpublished or ongoing trial data will also be searched from the following clinical trial registries: The Chinese clinical registry, the National Institutes of Health clinical registry Clinical Trials, the Australian New Zealand Clinical Trials Registry, the International Clinical Trials Registry Platform.

### 2.7 Data collection and analysis

#### 2.7.1 Selection of studies

**Figure 1:**
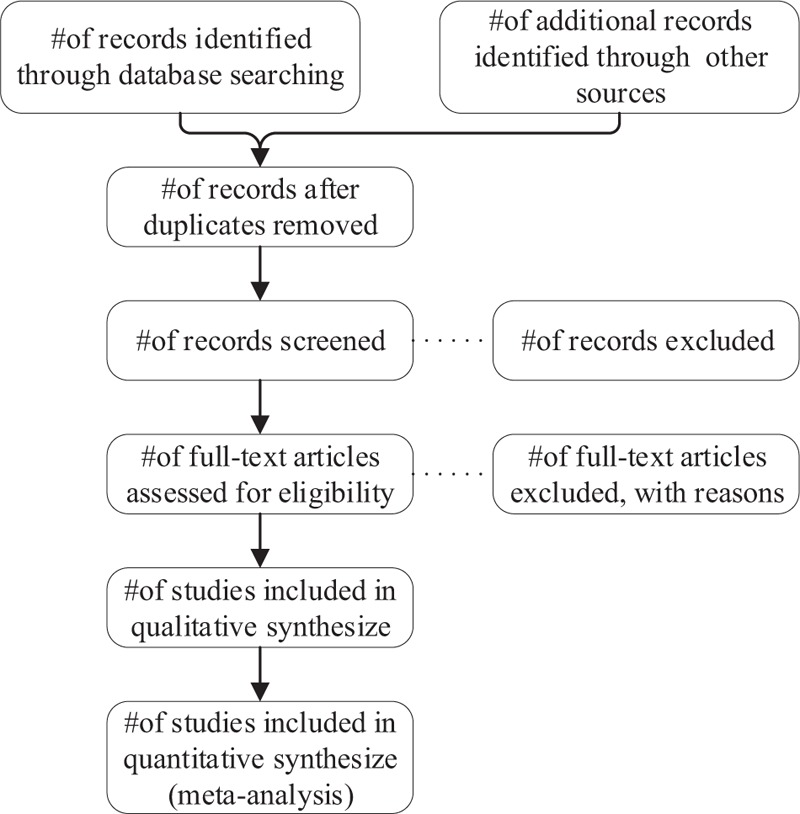
Illustrate the process of studies selection.

The Endnote software (version X9) will be used to manager the studies of electronic searches and obtained from other sources. First, we will getting rid of the duplicates according authors, title, and abstract (same content in different languages or different published forms, or 2 articles wrote the same trial from different aspects), the titles and abstracts will be screened independently by 2 reviewers for potentially qualified studies and exclude studies that are not content with selection criteria. If reviewers cannot identify the studies based on their titles and abstracts, them will screen the full text. When reviewers have an inconsistent opinion, they will be resolved through discussion. If no agreement is reached, a third reviewer will be consulted. The process and results of studies selection will be presented in a flow chart with Figure 1.

#### 2.7.2 Data extraction and management

We will confirmed a standard data extraction form using excel 2016 before data extraction, which will including the following information: basic information (including year of publication, the first author, publication source, etc), characteristic of trial (design of the study, number of groups and participants, method of randomization, blinding, method of analysis, objectives of the study, etc), participants (age, gender, ethnicity, country, diagnosis, duration, etc), interventions and controls (method of the intervention, time of intervention, number of treatment, frequency of treatment, duration of a session, name and type for control, additional treatment, etc), outcome measurements (primary outcome and secondary outcome, timeline for assessment, length of follow-up, etc), results (mean, standard deviation, observed events after intervention, total sample size, etc) and so on. Two reviewers will cross-check the results after extraction. If the different is consist, they will solve it by discussion among all the reviewers. The third reviewer will check the data entered to ensure the consistency and correct data entry errors.

### 2.8 Assessment of risk of bias in included studies

Two reviewers will use the Cochrane Collaboration's tool to evaluate the quality of the included trials. Six aspects (randomly generated sequence number, allocation concealment, blinding of participants and personnel, blinding of outcome assessment, incomplete outcome data, selective reporting, and other bias when required) will be assessed.^[41]^ The trial will be rated as high, low risk, or unclear of bias for each aspect. A trial that is rated high risk of bias in one or more aspects will be graded as ‘high risk’, while a low risk of bias in all aspects will be graded as ‘low risk’. If there is an unclear risk of bias for all main aspects, the trial will be rated as ‘unclear risk’. The rating results will be cross-checked and discrepancies resolved through discussions among all reviewers.

### 2.9 Measures of treatment effect

All data will be synthesized using RevMan5.2 or STATA software. The risk ratio/odds ratio with a 95% confidence intervals (CIs) will be presented the results of dichotomous data analysis, while continuous outcomes will be investigated by using the mean difference/the standardized mean difference with 95% CIs.

### 2.10 Dealing with missing data

We will contact the authors for included studies with missing data to get original data. If the authors cannot be contacted or the missing data is lost, the study will be excluded and the remaining studies will be synthesized.

### 2.11 Assessment of heterogeneity

The statistical heterogeneity will be assessed by chi-squared (*X*^2^) in forest plot using RevMan V.5.2 and a *P* value of less than .10 will be considered significant, according to the Cochrane Handbook.^[42]^ Moreover, we will quantify the impact of the statistical heterogeneity on the meta-analysis via calculating the *I*^2^ value using RevMan V.5.2. A rough guide to the interpretation of *I*^2^ is as follows: 0% to 40%: might not be important; 30% to 60%: may represent moderate heterogeneity; 50% to 90%: may represent substantial heterogeneity; 75% to 100%: considerable heterogeneity.^[42]^ Besides, the importance of the observed value of *I*^2^ depends on 2 aspects as follows:

(1) magnitude and direction of effects and
(2) strength of evidence for heterogeneity (eg, *P*-value from the *X*^2^ test, or a confidence interval for *I*^2^).^[42]^

### 2.12 Data synthesis

Before synthesizing the data, we will unify the units of each outcome from different trials, depending on the International System of Units. And then, clinical data will be imported into RevMan software (V.5.2) to perform data synthesis. Data will be synthesized and analyzed when the *I*^2^ < 75% coming from the heterogeneity test. We will use the fixed effects model to the pooled data when the heterogeneity tests show slight or no statistical heterogeneity in these trials (the *I*^2^ value is no less than 40%). The random effects model will be used for data synthesis when significant heterogeneity is detected (the *I*^2^ ≥40%, <75%). If there is considerable heterogeneity in the trials, meta-analysis will not be performed. In this case, we will try to identify the source of heterogeneity from both clinical and methodological aspects and a narrative, the qualitative summary will be provided.

A funnel plot will be generated to observe the reporting bias when more than 10 trials are included.

### 2.13 Subgroup analysis and meta-regression

If enough trials are included, we will explore the following potential sources of heterogeneity using STATA software with subgroup analyses or meta-regression from the variations in the characteristics of the trial participants, acupuncture treatments, sample size, methodological, missing data, and so on.

### 2.14 Sensitivity analysis

Sensitivity analysis will be used to check the stability of the primary decision made in the review process. Several decision nodes will be considered within the process of the systematic review, such as small sample size studies, methodological weaknesses and missing data. The results of the sensitivity analysis will be presented in summary tables. The risk of bias in the review process, as indicated by the results of the sensitivity analysis, will be discussed.

### 2.15 Evidence quality evaluation

The grading of recommendations assessment, development and evaluation system (GRADE) system will be used by 2 reviewers independently to assess the quality of evidence for each outcome.^[43]^ Evidence quality will be rated as ‘high’, ‘moderate’, ‘low’ or ‘very low’ according to the GRADE rating standards. The quality of evidence of a specific study will be assessed according to the risk of bias, inconsistency, indirectness, imprecision, publication bias, large effect, dose response, and all plausible confounding.^[43,44]^ A summary of findings table will be generated and included in the final report.^[44]^

### 2.16 Ethics and dissemination

This review does not require ethical approval due to data that we will not endanger the individual's privacy or compromise their rights. The results of a review that provide systematically view and evidence of AM for RLS will also give implication for clinical practice and further research, and the founding of this study may be published in a peer-reviewed journal or distributed at relevant conferences.

## Author contributions

Zhijun Huang conceived the review protocol and drafted the manuscript. Qingqing Cao, Wenchun Zhang revised the study design. Zhijun Huang, Qingqing Cao, Zhouhang Wu and Jiankun Ren participated in the design of the search strategy and data extraction data set. Qingqing Cao, Zhijun Huang, and Wenchun Zhang formed the data synthesis and analysis plan. In practice, Qingqing Cao and Zhijun Huang will monitor each procedure of the review and are responsible for the quality control. All authors have read and approved the publication of the protocol.

Zhijun Huang orcid: 0000-0002-9112-306X.
